# Topics of Public Interest

**Published:** 1909-05

**Authors:** 


### TOPICS OF PUBLIC INTEREST.

### Nutritive and Medicinal Value of Alcohol

### The Committee of Fifty and its Work.

A FEW persons have assumed to question the efficacy and value of alcohol as a remedial agent. The subject is an old one, and recent attacks on the therapeutic value of alcohol have been largely undertaken in sympathy with, if not in furtherance of, efforts to correct an evil of social life by the entire inhibition of alcohol. When an effort is made to stop abuses of alcohol through the entire suppression of the thing abused, there is naturally a desire to secure what is termed scientific authority for it.

Recent unfavorable discussions of the effects of alcohol and of its therapeutic value merely show the conclusions of one or two investigators, who may be considered as in the field to prove a case, like an expert in court. Those who know the other side through experience and practice constitute about the whole mass of mankind residing in the portion of the world called civilised.

The most vigorous characters in history have been temperate users of alcohol in one form or another. Some of the greatest, like Alexander of Macedon, were not temperate.

The recent unfavorable expert testimony shrinks to insignificance beside the exhaustive investigations of the Committee of Fifty and its conclusions. The reports of this great body of unbiased investigators have not had popular circulation, chiefly because of the great extent and the lack of public interest. For in reality the committee reported technically what nearly every intelligent person knew, but not in a technical way. The reports show conclusively that alcohol in suitable quantity acts as food.

"The Committee of Fifty" was organised in 1893 and headed by Hon. Seth Low, as president, Charles Dudley Warner as vice-president, and Professor Francis G. Peabody, D. D., of Cambridge, Mass., secretary.

Among the distinguished chemists connected with the investigations were Professor W. O. Atwater, Wesleyan University, Middletown, Conn.; Professor H. P. Bowditch, Harvard Medical School; Professor R. H. Chittenden, Sheffield Scientific School, of Yale University.

The committee included bishops and clergymen of several denominations, with business men, lawyers and authors, and the presidents of several universities.

After twelve years of careful inquiry, the work being divided among sub-committees, the conclusions were published in sections, as the committees rendered reports, in 1903-5. A summary of the conclusions of the committee was published in 1905, the introduction to the volume being written by Rev. Professor Francis G. Peabody.

Charles Dudley Warner wrote in Harper's Magazine, February, 1897, "That the prime business of the committee was not the expression of opinion or the advancing of one theory or another, but strictly the investigation of facts."

During the twelve years up to 1905 the committee had been in general session seventeen times, and its work is not yet finished.

For the purpose of correcting current misstatements as to the nature and effects of alcohol, some of the conclusion of the sub-committee of the committee of fifty on "The Physiological Aspects of the Liquor Problem" are briefly quoted in this paper. The chairman is Professor W. O. Atwater and the other members are John S. Billings, H. P. Bowditch, R. H. Chittenden and W. H. Walsh.

The most important experiments and investigations by this committee were conducted in the laboratories of physiological chemistry at Wesleyan University and Yale University. The experiments at Wesleyan under Professor Atwater were exhaustive and costly. The results were stated in two articles by Professor Atwater in Harper's Magazine for October and November, 1900, five years before the publication of the summary of the committee's work to which reference has been made.

Professor Atwater's first article in Harper's Magazine, October, 1900, was devoted to "The Nutritive Value of Alcohol," and somewhat extended reference to medicinal use especially in cases of critical illness.

Professor Atwater writes: "Is alcohol food? Has it a nutritive value? * * * The two chief functions of food are to furnish material for the formation of the tissues of the body, and to yield energy for warmth, and for muscular and other work. * * * The body uses the stored materials for food as it does the parts of the food which are not stored, but which are consumed as they are eaten. Alcohol contains no nitrogen, and, therefore cannot build tissue; it is rather to be classed with the fats and carbo-hydrates and if it has any food value, this must be as fuel. * * * When a man drinks wine, beer, brandy, or other liquor, is the alcohol oxidised? * * * I have found some seventeen investigations of sufficient importance to seem worthy of special consideration. The earliest were in 1846. The results of all but two were interpreted by their authors as indicating that alcohol is burned in the body." The most conclusive experiments to show that alcohol is burned in the body as fuel were by Anstie in England in 1865-1874.

Professor Atwater proceeds: "Since the work of Anstie the question has been how completely is alcohol burned?" Experiments in Bonn University, Germany, 1875-1883, by Drs. Binz and Bodlander, are detailed. These experimenters found that as a rule 95 per cent. or more of alcohol taken into the body in moderate quantity was oxidised. In 1871, Strassman in Berlin, found that with large doses of alcohol "only 6 or 7 per cent. escaped oxidation."

Professor Atwater continuing, says: "In the late experiments under my own direction, described beyond, alcohol was given in smaller doses, and especial pains were taken to measure accurately the amounts which left the body by various channels. In these cases less than 2 per cent. has generally escaped oxidation. * * * * As the experiments with alcohol have become more accurate, the proportion actually oxidised has appeared larger and larger. When taken in small quantities, say one or two glasses of wine or a glass of whiskey at a time, the alcohol has been found to be burned at least as completely as bread or meat."

Having shown that alcohol is oxidised in the body, Professor Atwater proceeds to describe his exhaustive laboratory experiments with several men to ascertain if alcohol supplies working energy, if substituted in part for a regular diet. His apparatus was such as would show the results of regular foods and of alcohol. He says of these experiments: "The average energy given off from the body was in the experiments without alcohol 2.949 calories, in those with alcohol 2,952 calories per day. Neither my associates nor myself can find in them any indication of considerable extra radiation of heat under the influence of alcohol, nor can we find anything which seems to us to justify the conclusion that the energy of the diet without alcohol was used more economically than that of the diet with alcohol. * * * * So far as I know, the experimenters whose work is most generally accepted as reliable are practically agreed that alcohol by being burned in the body protects fat from consumption." * * * In our own experiments the men lost on an average no more fat in the experiments with alcohol than in the corresponding ones with ordinary diet, and stored as much."

Of the medicinal and sustaining effects of alcohol in disease Professor Atwater says. "In talking with physicians about this subject I have been much impressed by the frequent and emphatic statements of their experience in administering alcohol to patients in forms of disease when the bodily activities are at a low ebb. They tell me that they frequently find that people in such condition will take without intoxicating effects quantities of alcohol which would under ordinary circumstances produce drunkenness. They say, furthermore, that there are many cases in which the bodily functions are maintained and life is even saved by alcohol when ordinary food could not be endured. * * * Dr. Anstie gives the details of a number of interesting cases of this kind, which he evidently studied with great care. From the standpoint of the physiological chemist, this effect of alcohol would seem entirely natural. The bodily functions are weakened and the power of digestion impaired. While the patient is lying still, the labor required of the muscles is not large, and the chief need is fuel to carry the body through the time of stress. What is wanted is a material which will not have to be digested, can be easily absorbed, is readily oxidised and will supply the requisite energy. I know of no other material which would seem to meet these requirements so naturally and so fully as alcohol. It does not require digestion, is absorbed by the stomach, and presumably by the intestines with great ease. Outside the body it is oxidised very readily, within the body it appears to be quickly burned, and supplies a large amount of energy."

The report of the sub-committee on the "physiological aspects" says of alcohol and digestion: "When alcoholic fluids are taken into the stomach in not too large quantities, there is first a direct stimulation, leading to the rapid secretion of a powerful gastric juice. This is followed by a more or less rapid absorption of the alcohol, accompanied in turn by an indirect or secondary stimulation of gastric secretion. * * * Regarding salivary digestion, alcohol and alcoholic beverages when taken into the mouth produce a direct stimulating effect upon the secretion of saliva." * * * Alcohol taken in moderate quantities produces effects on nutrition similar to those produced by the starches, sugars and fats in ordinary food, in that it is oxidised in the body and yields energy for warmth and possibly for muscular work. Roughly speaking, four grains of alcohol will yield the same amount of energy as seven grains of sugar, starch or protein, or three grains of fat."

Of the frequent designation of alcohol as "a poison" when taken in excess, the committee report says: "There is no substance which is always and everywhere a poison. The term is relative; conditions and circumstances of various kinds must always enter into its conception. No one would maintain that a cup of delicately flavored tea is in any sense injurious or poisonous to the average adult and yet caffeine, the active principle of this cup of tea, is a poison as surely as alcohol. The term poison belongs with equal propriety to a number of other food accessories, as coffee, pepper, ginger, and even common salt."

While admitting frankly the injurious effects of excessive use of alcohol in causing disease, the committee says of some diseases usually attributed to alcoholic excesses: "It is important to know that the immoderate drinking of alcoholic liquors may be the first symptom of some chronic disease which, when later recognised, is erroneously ascribed to alcohol as the cause. It is known that many of the mental and nervous disorders of alcoholism, attributed to the toxic action of alcohol are. nevertheless, dependent in a large measure on an underlying defective constitution, as an excessive indulgence in alcohol rarely produces certain of these disorders in persons of normal constitution."

This brief abstract of the chief results of an exhaustive inquiry as to some effects of alcohol are submitted with confidence that much of current error and misrepresentation will be corrected. The Committee of Fifty is a very cautious body; for it has among its members total abstainers and prohibitionists; but all are honest men. So the truth in regard to a most useful agent has been barely told.

---

Institutional Work for Young Physicians.—Wm. P. Spratling of Baltimore, Md., describes the positions available for young physicans in connection with asylums and sanatoriums. The State institutions for the insane afford very desirable positions with a salary beginning with $600 and maintenance and increasing by length of service and examinations for promotion up to $4,500. Boards of Health and municipal departments also afford positions useful for those who do not wish to enter general practice at once, or need assistance through a few years. Sanatoria and public health resorts also employ resident physicians, who generally receive good salaries.—*Medical Record,* April 24, 1909.

